# Pigeons as overlooked vectors: investigating *Campylobacter* carriage across different management systems

**DOI:** 10.1186/s12917-026-05479-8

**Published:** 2026-04-30

**Authors:** Aleksandra Kobuszewska, Paulina Przyborowska, Beata Wysok

**Affiliations:** Department of Veterinary Public Health, Faculty of Veterinary Medicine, University of Warmia and Mazury in Olsztyn, Olsztyn, Poland

**Keywords:** *Campylobacter*, pigeons, AMR, genetic diversity, flaA short variable region

## Abstract

**Background:**

*Campylobacter* spp. are among the leading bacterial pathogens responsible for foodborne illness worldwide, yet the role of pigeons as reservoirs remains insufficiently investigated compared with poultry and livestock.

**Results:**

In this study, we examined 450 pigeons from three distinct groups—backyard, racing, and ornamental flocks—to assess the prevalence, virulence gene repertoire, genotypic diversity, and antimicrobial resistance (AMR) of *Campylobacter* isolates. The overall prevalence reached 29.1%, with the highest rates observed in backyard pigeons (37.3%), followed by racing (28.7%) and ornamental (21.3%) birds. Species identification revealed that *C. jejuni* predominated (85.5%), while *C. coli* accounted for 14.5% of isolates. Virulence genes were widely distributed, with universal presence of *flaA*, high frequencies of adhesion and invasion markers (*cadF*, *ciaB*), and the complete *cdtABC* cluster in 72.5% of isolates, whereas GBS-associated genes were less frequent. Sequencing of the *flaA-SVR* region revealed 48 alleles, several of which were previously associated with wild birds, livestock, or poultry, demonstrating genetic overlap across ecological compartments. Antimicrobial resistance was widespread, with tetracycline (64.1%) and fluoroquinolone resistance (ciprofloxacin 54.2%, erythromycin 49.6%) being most common, and gentamicin resistance remaining less frequent (14.5%). Multidrug resistance (MDR) was detected in 22.1% of isolates, with backyard pigeons harbouring the greatest proportion of MDR strains.

**Conclusions:**

These findings indicate that pigeons maintain genetically diverse and virulent *Campylobacter* populations with relevant AMR profiles. The high prevalence of these pathogens in pigeons, often exceeding that reported in wild birds, combined with pigeons’ close contact with humans and domestic animals, underscores their role as an important yet underappreciated reservoir in the epidemiology of campylobacteriosis.

## Background

Campylobacteriosis, primarily caused by *Campylobacter jejuni* and *C. coli*, is currently the most frequently reported zoonotic bacterial infection in the European Union. This zoonosis is responsible for substantial public health and economic burdens worldwide [1–4]. Although poultry and livestock have been extensively studied as major reservoirs [5–9], free-living wild birds remain an important source of *Campylobacter* infections in humans [10–12]. Among them, urban pigeons (*Columba livia domestica*) have been recurrently examined as potential carriers due to their abundance in metropolitan areas, close contact with human populations, and the ease with which they may contaminate public spaces [13–17].

For centuries, pigeons have been bred for communication purposes as postal messengers, as well as for sport and ornamental display [18–20]. While their utilitarian importance has diminished in modern times, pigeon breeding remains a popular practice worldwide, sustained by enthusiasts and hobbyists [21–23]. At present, pigeon keeping has taken several distinct forms, including ornamental pigeon breeding, pigeon racing as a sport, and small-scale backyard flocks [24]. Each of these systems is characterised by unique management practices, husbandry conditions, and interactions with humans and other animals, which may influence the epidemiology of infectious agents such as *Campylobacter* [25–27]. Racing pigeons, for example, are trained to travel long distances and participate in competitions that involve contact with other birds across regional, national, or even international borders [25, 28, 29]. This mobility increases the risk of pathogen acquisition and dissemination over large geographic ranges [30–32]. In contrast, ornamental pigeons are usually maintained in aviaries or enclosed lofts, with limited direct exposure to wild birds but frequent close contact with humans who handle them. Backyard pigeons represent a more heterogeneous group as they are often kept in semi-open systems where they may interact freely with domestic poultry, livestock, companion animals, or even wildlife [33–36]. Such varied husbandry conditions could create diverse ecological interfaces for pathogen circulation and persistence, yet these pigeon populations have rarely been included in systematic microbiological investigations.

The role of pigeons as potential reservoirs of *Campylobacter* has been addressed only sporadically by researchers, and even less attention has been devoted to the antimicrobial susceptibility of isolates originating from these birds [10, 11, 37]. Unlike intensively farmed poultry, pigeons are not routinely subjected to antimicrobial treatments, yet their diverse management systems expose them to indirect selective pressures. Backyard pigeons may come into contact with antimicrobials used in nearby livestock farms; racing pigeons are regularly transported across regions with different levels of environmental contamination, and ornamental pigeons can acquire resistant strains through close interactions with other avian species kept in private collections [38–40]. This allows resistant *Campylobacter* strains to circulate largely unnoticed, independently of the conventional food-production chain [41, 42]. Therefore, investigating resistance patterns in pigeon-derived isolates may help identify overlooked pathways involved in the persistence and dissemination of antimicrobial resistance (AMR).

In addition to prevalence estimates, the genetic diversity and virulence-associated traits of pigeon-associated *Campylobacter* also remain insufficiently explored. While studies in poultry have extensively profiled genes linked to adhesion, invasion, and toxin production, the presence and distribution of these markers in pigeon-derived isolates are still poorly understood. Similarly, sequencing-based approaches such as *flaA* short variable region (*flaA*-SVR) typing have been applied to trace the evolutionary dynamics of poultry and wild-bird strains, but comparable data for pigeon populations are almost entirely lacking [43–48]. These molecular features should be investigated to determine whether pigeon-associated *Campylobacter* strains possess attributes that may facilitate zoonotic transmission or explain unique host adaptations that are distinct from those described in intensively farmed birds.

From a public health perspective, this knowledge gap is striking, as the poultry sector has been extensively studied and subjected to regulatory frameworks aimed at *Campylobacter* control, whereas pigeon flocks remain largely unmonitored [49, 50]. This lack of data leaves an open question about their contribution to zoonotic transmission cycles, especially given their proximity to humans in rural, peri-urban, and hobbyist settings. Moreover, the role of pigeons as potential bridge hosts between wild birds, domestic poultry, and humans remains poorly understood [51–53].

Given that campylobacteriosis continues to be regarded as an emerging and insufficiently addressed problem, identifying all potential sources of infection is of crucial importance [54–57]. In this context, non-urban pigeons may represent an overlooked but epidemiologically relevant reservoir, as their diverse husbandry systems, frequent interactions with humans, and potential contact with both domestic and wild animals create conditions conducive to the acquisition, maintenance, and transmission of *Campylobacter* strains across ecological boundaries [58–61]. Addressing this gap aligns with the core tenets of the One Health approach, which underscores the interconnectedness of human, animal, and environmental health [62, 63]. Expanding *Campylobacter* surveillance to include ornamental, racing, and backyard pigeons not only advances our understanding of pathogen ecology but also contributes to the development of more comprehensive public health strategies aimed at reducing zoonotic risks.

In this study, the prevalence of *Campylobacter* spp. was assessed in three groups of non-urban pigeons—ornamental, racing, and backyard flocks—to elucidate their potential role as reservoirs of this pathogen. By comparing populations kept under distinct husbandry conditions, we aimed to generate new insights into underexplored sources of infection and to strengthen the evidence base relevant to the One Health framework for campylobacteriosis prevention and control. The existing literature remains disproportionately centred on feral, city-dwelling pigeons, leaving other pigeon populations comparatively neglected. Therefore, this study aimed to determine the prevalence of *Campylobacter* spp. in ornamental, racing, and backyard pigeons, to characterize the virulence gene profiles and genetic diversity of the isolates, and to assess their antimicrobial resistance patterns.

## Methods

### Sampling 

A total of 450 cloacal swab samples were collected between January and December 2024 from pigeons grouped into three distinct categories: racing pigeons (*n* = 150), ornamental pigeons (*n* = 150), and backyard flocks (*n* = 150). All sampled birds belonged to domestic pigeon populations (*Columba livia f. domestica*). Samples were collected at regular intervals throughout the year, with flocks selected according to availability and owner consent. The sample size was determined using a standard formula for prevalence studies, assuming an expected prevalence of 50%, a 95% confidence level, and a 5% margin of error. This approach yielded a minimum required sample size of approximately 384 samples. To allow balanced comparisons among the three pigeon categories and to account for potential sample loss or invalid results, the total sample size was increased to 450, with 150 pigeons sampled from each group. The groups differed in management practices. Racing pigeons were actively engaged in competitive flights, while ornamental pigeons were primarily housed in lofts or aviaries. Backyard pigeons were maintained under semi-open conditions with intermittent interaction with other domestic animals, and they were not part of commercial meat production systems, but were kept mainly for hobby, breeding, or small-scale domestic purposes. All sampled pigeons were clinically healthy at the time of sampling and did not exhibit visible signs of disease, including gastrointestinal symptoms. The samples were collected in north-eastern Poland, within the Warmian–Masurian region, in the area spanning approximately 53.7–54.2° N and 20.3–21.1° E, including locations around Olsztyn, Lidzbark Warmiński, and Orneta. Cloacal swabs were collected as part of standard routine handling procedures to minimise stress and immediately placed in Amies transport medium with charcoal (Deltalab S.L., Barcelona, Spain). Samples were transported under refrigeration to the laboratory for subsequent microbiological analysis.

### Isolation 

Isolation was carried out according to the EN ISO 10272-1:2017 [64] standard method for the detection of *Campylobacter* spp. Each swab was transferred into 9 mL of Bolton broth (Oxoid, UK) and incubated at 37 °C for 4 h, followed by 41.5 °C for 44 ± 4 h under microaerophilic conditions (5% O₂, 10% CO₂, 85% N₂). A loopful of enrichment culture was then streaked onto modified charcoal cefoperazone deoxycholate agar (mCCDA, Oxoid, UK) and Karmali agar (Oxoid, UK). Plates were incubated for 24–48 h under identical microaerophilic conditions. Presumptive *Campylobacter* colonies were selected based on characteristic morphology (small, moist, greyish colonies with a shining or spreading appearance). Each isolate was subjected to standard confirmatory tests, including Gram staining, motility assessment, oxidase reaction, and absence of aerobic growth at 25 °C. Single colonies were subcultured once and stored at -80 °C in defibrinated horse blood (Oxoid, UK) supplemented with glycerol (80:20 v/v).

### Identification 

The species-level identification of recovered isolates was performed using PCR. Colonies were grown on blood-supplemented Columbia agar (Oxoid, UK) resuspended in sterile water and centrifuged at 13,000 × g for 1 min. DNA was extracted using the Genomic Mini Kit (A&A Biotechnology, Gdańsk, Poland) according to the manufacturer’s protocol. DNA quality and concentration were measured spectrophotometrically, and species-specific PCR assays were applied to differentiate between *C. jejuni* and *C. coli*. Primer sequences are provided in Table 1.

**Table 1:** List of primers used in the study for the amplification of virulence genes

Target Gene	Sequences (5′–3′)	Annealing temperature (°C)	References
*16 S rRNA* for *Campylobacter* spp.	F-ATCTAATGGCTTAACCATTAAAC R-GGACGGTAACTAGTTTAGTATT	55	[65]
*mapA* for *C. jejuni*	F-CTATTTTATTTTTGAGTGCTTGTG R-GCTTTATTTGCCATTTGTTTTATTA	55	[65]
*ceuE* for *C. coli*	F-AATTGAAAATTGCTCCAACTATG R-TGATTTTATTATTTGTAGCAGCG	55	[65]
*flaA*	FAATAAAAATGCTGATAAAACAGGTG R-TACCGAACCAATGTCTGCTCTGATT	53	[66]
*flaA-SVR*	F-CTATGGATGAGCAATT(AT)AAAAT R-CAAG(AT)CCTGTTCC(AT)ACTGAAG	50	[67]
*cadF*	F-TTGAAGGTAATTTAGATATG R-CTAATACCTAAAGTTGAAAC	45	[68]
*iam*	F-GCGCAAAATATTATCACCC R-TTCACGACTACTATGCGG	50	[69]
*ciaB*	F-TGCGAGATTTTTCGAGAATG R-TGCCCGCCTTAGAACTTACA	62	[70]
*pldA*	F-AAGCTTATGCGTTTTT R-TATAAGGCTTTCTCCA	47	[66]
*cdtA*	F-CCTTGTGATGCAAGCAATC R-ACACTCCATTTGCTTTCTG	55	[66]
*cdtB*	F-CAGAAAGCAAATGGAGTGTT R-AGCTAAAAGCGGTGGAGTAT	55	[66]
*cdtC*	F-CGATGAGTTAAAACAAAAAGATA R-TTGGCATTATAGAAAATACAGTT	53	[66]
*pebA*	F-GCTCTAGGTGCTTGTGTTGC R-GTAGTTGCAGCTTGAGCCAC	50	[71]
*porA*	F-TCAACTGGACACTTGAAGGTGC R-CCACCATATACGAAGTCAGCACC	52	[71]
*jlpA*	F- GCACACAGGGAATCGACAGC R-AAATGACGCTCCGCCCATTAAC	52	[71]
*virB11*	F-TCTTGTGAGTTGCCTTACCCCTTTT R-CCTGCGTGTCCTGTGTTATTTACCC	55	[66]
*cgtB*	F-TAAGAGCAAGATATGAAGGTG R-GCACATAGAGAACGCTACAA	52	[72]
*wlaN*	F-TGCTGGGTATACAAAGGTTGTG R-ATTTTGGATATGGGTGGGG	54	[73]

### Screening for virulence-associated genes 

PCR was also used to screen for a panel of virulence-associated genes, including adhesion (*flaA*,* cadF*,* jlpA*,* porA*,* pebA*), invasion (*ciaB*,* pldA*,* iam*,* virB11*), cytotoxicity (*cdtA*,* cdtB*,* cdtC*), and GBS-related markers (*wlaN*,* cgtB*). Each reaction was performed in a total volume of 50 µL containing 10× PCR buffer, dNTPs (final concentration of 200 µM), MgCl₂ (5 mM), thermostable Taq polymerase (Thermo Fisher Scientific, USA), primers (each with a final concentration of 0.1 µM), and ~ 120 ng of template DNA. Cycling conditions consisted of an initial denaturation step at 94 °C (2 min), 35 amplification cycles (94 °C for 1 min, primer-specific annealing for 1 min, 72 °C for 1 min), and a final extension step at 72 °C for 5 min. PCR products were separated on 2% agarose gels stained with ethidium bromide and visualised against a 100-bp molecular marker. Each assay included positive controls (*C. jejuni* ATCC 33291, *C. coli* ATCC 43478) and a negative control (nuclease-free water).

### Sequencing of flaA-SVR 

For molecular typing, the short variable region of *flaA* was amplified and sequenced using the previously described primers (Table 1). Amplicons were visualised, purified using a Clean-Up Kit (A&A Biotechnology, Poland), and sequenced by the Sanger method at Genomed (Warsaw, Poland). Forward and reverse reads were assembled and trimmed to a 321-bp consensus sequence using Vector NTI Express (Thermo Fisher Scientific, USA). Allelic numbers were assigned based on the PubMLST Campylobacter database. A maximum likelihood phylogenetic tree was constructed using MEGA X. Node support was evaluated using bootstrap resampling with 1000 replicates. Bootstrap values above 70% were considered to indicate strong support. The tree was visualised in iTOL v4. Genetic diversity indices were calculated using the Comparing Partitions tool (http://www.comparingpartitions.info). The obtained sequences were submitted to the GenBank database and received the following Accession Numbers PZ191485 - PZ191615.

### Antimicrobial susceptibility testing 

Antimicrobial resistance profiles were assessed using agar dilution MIC testing in accordance to EUCAST guidelines. The antimicrobials tested in this study were selected according to the recommendations of the European Food Safety Authority (EFSA) for harmonized antimicrobial resistance monitoring in *Campylobacter* spp., where erythromycin, ciprofloxacin, tetracycline, and gentamicin are included as key antimicrobials to assess resistance patterns relevant to both public health and veterinary surveillance. Inocula were standardised to 0.5 McFarland and diluted to ~ 10⁴ CFU/mL in Mueller–Hinton broth (Biomaxima, Poland). Using a Steers multipoint replicator, suspensions were inoculated onto Mueller–Hinton agar plates containing serial twofold dilutions of antimicrobials (range: 0.015–64 mg/L for E and CIP; 0.03–128 mg/L for TE and CN). Plates were incubated microaerobically at 41.5 °C for 24 h, and MIC endpoints were recorded as the lowest concentration inhibiting visible growth. Control strain *C. jejuni* ATCC 33,560 was included. EUCAST clinical breakpoints established for *Campylobacter* were applied to E, CIP, and TE, whereas CN interpretation followed the *Enterobacteriaceae* breakpoints. Strains resistant to ≥ 3 antimicrobial classes were classified as multidrug-resistant (MDR).

### Statistical analysis 

Data were analysed using Statistica v13.3 (StatSoft, Poland). Simpson’s Diversity Index (D) was calculated to quantify the genetic diversity of *flaA*-SVR alleles among *Campylobacter* isolates and to evaluate the degree of heterogeneity within the analyzed populations. Comparisons of antimicrobial resistance patterns and the prevalence of virulence-associated genes between pigeon groups were performed using the Chi-square test or Fisher’s exact test, as appropriate. To account for multiple comparisons, p-values were adjusted using the Bonferroni correction. A conservative approach was adopted to minimize the risk of type I error. A two-sided p-value of < 0.05 after correction was considered statistically significant.

## Results

### Isolation and species identification

*Campylobacter* spp. were recovered from 131 (29.1%) out of the total of 450 cloacal swabs. Carriage rates varied across groups, ranging from 21.3% in ornamental pigeons to 37.3% in backyard flocks, with racing pigeons showing intermediate values (28.7%). Species identification showed a clear predominance of *C. jejuni* (85.5%) over *C. coli* (14.5%) across all groups. At the same time, the distribution of *C. coli* differed across the tested categories. The relative proportion of *C. coli* was highest in racing pigeons (18.6%), compared with 12.5% in both backyard and ornamental birds (Table 2).

**Table 2:** Prevalence and species distribution of *Campylobacter* spp. isolated from three groups of pigeons

Group	Samples tested (*n*)	Campylobacter-positive		C. jejuni		C. coli	
		*n*	%	*n*	%	*n*	%
Backyard	150	56	37.3	49	32.7	7	4.7
Racing	150	43	28.7	35	23.3	8	5.3
Ornamental	150	32	21.3	28	18.7	4	2.7
Total	**450**	**131**	**29.1**	**112**	**24.9**	**19**	**4.2**

### Detection of virulence genes

Among adhesion-related genes, the *flaA* gene was identified as a universally conserved genetic marker across all examined isolates, with a prevalence of 100.0% (131/131). The *cadF* gene was the second most prevalent adhesion-associated gene, identified in 91.6% (120/131) of isolates. Its distribution was strongly influenced by species-specific and group-specific factors: it was more frequently detected in *C. jejuni* (94.6%, 106/112) than in *C. coli* (73.7%, 14/19). Among pigeon populations, *cadF* was most prevalent in backyard flocks (94.6%, 53/56) and ornamental pigeons (93.8%, 30/32), and slightly less prevalent in racing pigeons (86.0%, 37/43). Adhesion-related genes, including *porA* (74.8%, 98/131), *pebA* (81.7%, 107/131), and *jlpA* (68.7%, 90/131), were highly prevalent across the isolates (Table 3). A statistical analysis did not reveal significant differences in the distribution of adhesion-associated genes between pigeon groups (*p* > 0.05).

**Table 3:** Distribution of virulence-associated genes in *Campylobacter jejuni* and *Campylobacter coli* isolates from pigeons

Group	Species	flaA	cadF	jlpA	porA	pebA	ciaB	pldA	iam	virB11	cdtA	cdtB	cdtC	wlaN	cgtB
Backyard	*C. jejuni*	49/49 (100%)	47/49 (95.9%)	34/49 (69.4%)	36/49 (73.5%)	38/49 (77.6%)	44/49 (89.8%)	37/49 (75.5%)	18/49 (36.7%)	3/49 (6.1%)	36/49 (73.5%)	38/49 (77.6%)	37/49 (75.5%)	–	3/49 (6.1%)
	*C. coli*	7/7 (100%)	6/7 (85.7%)	3/7 (42.9%)	5/7 (71.4%)	4/7 (57.1%)	4/7 (57.1%)	3/7 (42.9%)	2/7 (28.6%)	1/7 (14.3%)	4/7 (57.1%)	4/7 (57.1%)	3/7 (42.9%)	1/7 (14.3%)	–
Racing	*C. jejuni*	35/35 (100%)	33/35 (94.3%)	22/35 (62.9%)	25/35 (71.4%)	27/35 (77.1%)	31/35 (88.6%)	23/35 (65.7%)	14/35 (40.0%)	3/35 (8.6%)	26/35 (74.3%)	28/35 (80.0%)	29/35 (82.9%)	–	5/35 (14.3%)
	*C. coli*	8/8 (100%)	7/8 (87.5%)	3/8 (37.5%)	5/8 (62.5%)	4/8 (50.0%)	5/8 (62.5%)	4/8 (50.0%)	3/8 (37.5%)	1/8 (12.5%)	5/8 (62.5%)	6/8 (75.0%)	5/8 (62.5%)	2/8 (25.0%)	1/8 (12.5%)
Ornamental	*C. jejuni*	28/28 (100%)	26/28 (92.9%)	19/28 (67.9%)	20/28 (71.4%)	23/28 (82.1%)	25/28 (89.3%)	21/28 (75.0%)	11/28 (39.3%)	2/28 (7.1%)	23/28 (82.1%)	22/28 (78.6%)	23/28 (82.1%)	–	4/28 (14.3%)
	*C. coli*	4/4 (100%)	4/4 (100%)	2/4 (50.0%)	3/4 (75.0%)	2/4 (50.0%)	2/4 (50.0%)	1/4 (25.0%)	2/4 (50.0%)	–	2/4 (50.0%)	2/4 (50.0%)	2/4 (50.0%)	–	–
Total	*C. jejuni*	112/112 (100%)	106/112 (94.6%)	75/112 (67.0%)	81/112 (72.3%)	88/112 (78.6%)	100/112 (89.3%)	81/112 (72.3%)	43/112 (38.4%)	8/112 (7.1%)	85/112 (75.9%)	88/112 (78.6%)	89/112 (79.5%)	-	12/112 (10.7%)
	*C. coli*	19/19 (100%)	17/19 (89.5%)	8/19 (42.1%)	13/19 (68.4%)	10/19 (52.6%)	11/19 (57.9%)	8/19 (42.1%)	7/19 (36.8%)	2/19 (10.5%)	11/19 (57.9%)	12/19 (63.2%)	10/19 (52.6%)	3/19 (15.8%)	1/19 (5.3%)

A complete adhesion gene cluster (*flaA–cadF–jlpA–porA–pebA*) was found in 66.4% (87/131) of the *Campylobacter* strains tested, with a prevalence ratio of 73.2% (41/56) in backyard pigeons, 62.5% in ornamental pigeons (20/32), and 60.4% in racing pigeons (26/43). Species-wise, a full adhesion pattern was observed in 69.6% (78/112) of *C. jejuni* isolates and in 47.4% (9/19) of *C. coli* isolates. These differences were not statistically significant between the analysed groups (*p* > 0.05).

The *ciaB* gene was the most prevalent invasion marker, identified in 85.5% (112/131) of all isolates. It was more prevalent in *C. jejuni* (92.0%, 103/112) than in *C. coli* (47.4%, 9/19). At the group level, *ciaB* was predominantly identified in ornamental pigeons (87.5%, 28/32), followed by backyard pigeons (85.7%, 48/56) and racing pigeons (81.4%, 35/43). The *pldA* gene was present in 73.3% (96/131) of isolates, predominantly in *C. jejuni* strains (75.9%, 85/112). At the group level, a higher prevalence was observed in ornamental and backyard pigeons (both at 75.0%) than in racing pigeons (67.4%). The *iam* gene was detected in 40.5% (53/131) of isolates, with a slightly higher prevalence in *C. coli* (52.6%, 10/19) than in *C. jejuni* (38.4%, 43/112). Notably, the distribution of the *iam* gene differed significantly between pigeon groups, with a higher prevalence observed in backyard pigeons compared to racing pigeons (*p* = 0.037). The *virB11* gene was the rarest invasion gene, identified in 8.4% (11/131) of isolates, predominantly in *C. jejuni* (8/11; 72.7%). A complete invasion gene pattern (*ciaB-pldA-iam-virB11*) was observed in 11 isolates (three *C. coli* and eight *C. jejuni* strains), which were evenly distributed among backyard, racing, and ornamental pigeons. No statistically significant differences were observed between groups for the remaining invasion-associated genes (*p* > 0.05).

Genes encoding cytolethal distending toxins (*cdtA*,* cdtB*,* cdtC*) were detected at broadly comparable frequencies among the examined isolates. The *cdtB* gene was most prevalent, occurring in 79.4% (104/131) of strains, followed by *cdtA* in 76.3% (100/131) and *cdtC* in 75.6% (99/131) of isolates. These determinants were predominantly associated with *C. jejuni*, with 82.1% (92/112) of isolates carrying at least two genes of the *cdt* cluster. In contrast, nearly half of the *C. coli* strains (47.4%, 9/19) lacked any cytotoxicity-associated markers. A group-level analysis did not reveal clear predominance within any specific cohort; instead, the distribution varied by individual gene. Notably, *cdtA* was most prevalent in isolates from ornamental pigeons (81.3%; 26/32), whereas *cdtB* and *cdtC* were most frequently detected in isolates from racing pigeons, with prevalence rates of 81.4% (35/43) and 83.7% (36/43), respectively. Given the high overall prevalence of cytotoxicity-associated genes, a significant proportion of isolates exhibited the complete *cdtA–cdtB–cdtC* gene cluster. The complete cluster was present in 72.5% (95/131) of isolates and was most prevalent in racing pigeon isolates (34/43; 79.1%). Statistical analysis demonstrated a significant difference in the distribution of the *cdtC* gene, with higher prevalence in backyard pigeons compared to racing pigeons (*p* = 0.022), while no significant differences were observed for *cdtA* and *cdtB* (*p* > 0.05).

The genes associated with Guillain-Barré syndrome were less prevalent than other genetic markers. Specifically, the *wlaN* gene was detected in 17.6% (23/131) of isolates, whereas the *cgtB* gene was identified in 15.3% (20/131) of the analysed strains. The *wlaN* gene was found only in a single isolate of *C. coli*, while the *cgtB* gene was identified only in *C. jejuni* isolates. Notably, these genes were most prevalent in isolates from racing pigeons, with detection rates of 23.3% for *wlaN* and 18.6% for *cgtB*. Conversely, ornamental and backyard pigeon isolates showed the lowest prevalence (12.5%, 4/32 in both groups). No statistically significant differences were observed between pigeon groups for *wlaN* and *cgtB* (*p* > 0.05).

### *flA-SVR* sequencing

The analysis of *flaA*-SVR sequences from 131 *Campylobacter* isolates revealed a total of 48 distinct alleles, indicating considerable genetic heterogeneity. The most prevalent allele was 93, detected in 9 out of 131 isolates (6.9%), followed by allele 199 in 8/131 isolates (6.1%), and alleles 383, 419, 22, 67, 80, 111, and 766, each identified in 5/131 isolates (3.8%). Additional recurrent alleles included alleles 262, 294, and 58, each present in 4/131 isolates (3.1%). The remaining alleles exhibited low frequencies, with 32 out of 48 alleles (66.7%) being unique to single isolates.

Species-level divergence was evident in the *flaA*-SVR allele profiles. *Campylobacter jejuni* exhibited higher overall diversity, with alleles 93 (9/112; 8.0%) and 199 (8/112; 7.1%) being the most common. In contrast, *C. coli* isolates clustered around a few predominant variants, notably 262 and 294 (4/19; 21.1%) and 1376 (3/19; 15.8%).

Genetic differentiation was also evident in pigeons from different management systems. Alleles 199 (6/56; 10.7%), 111 (3/56; 5.4%), and 766 (3/56; 5.4%) were most frequently identified in backyard pigeons (*n* = 56). Alleles 93 (5/43; 11.6%), 294 (3/43; 7.0%), and 383 (2/43; 4.7%) were predominant in racing pigeons (*n* = 43). In turn, the highest prevalence of allele 419 (4/32; 12.5%) was observed in ornamental pigeons (*n* = 32), followed by alleles 67 and 383 (3/32; 6.3% each).

**Fig. 1:**
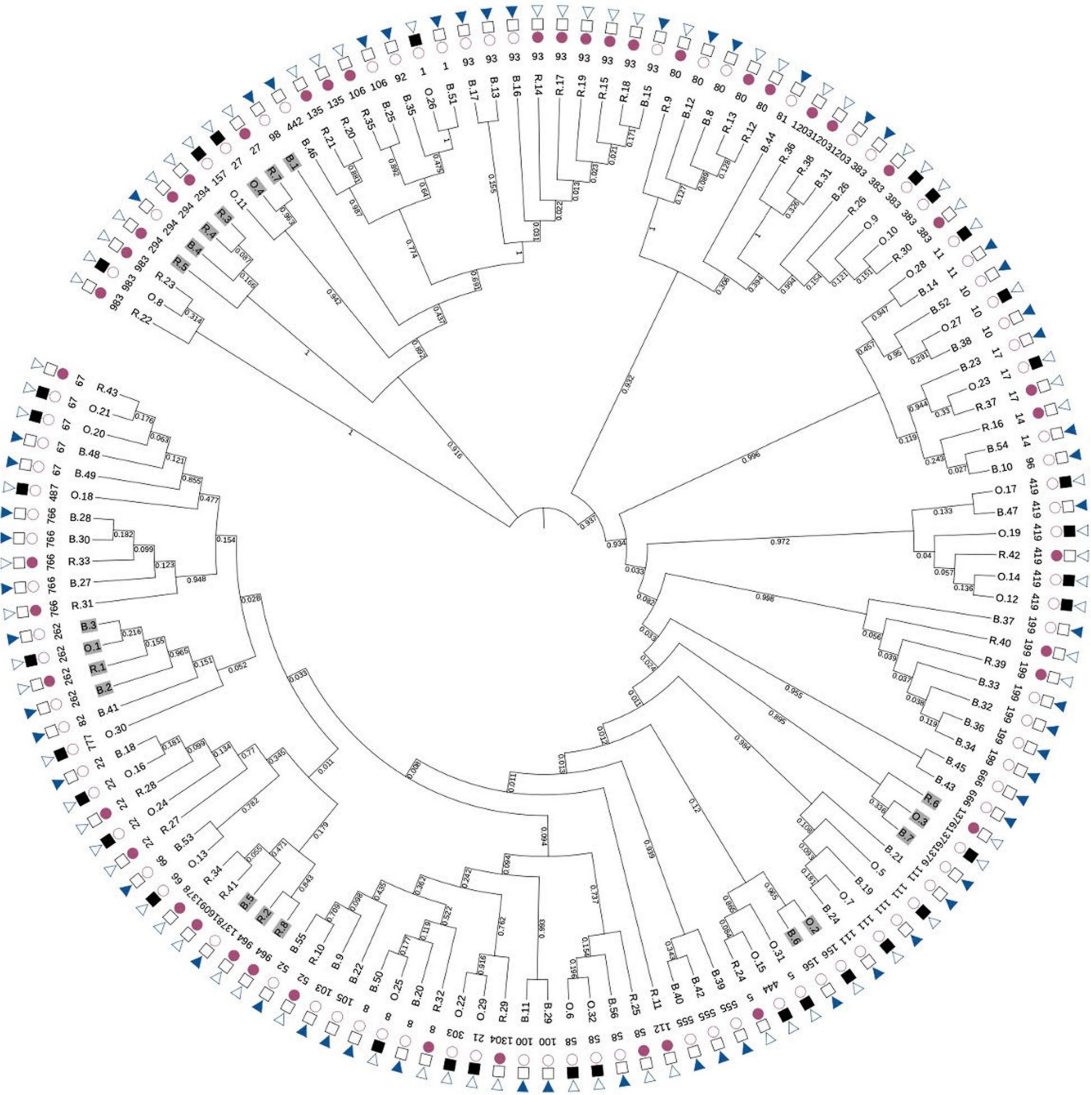
*Campylobacter* isolates obtained from pigeons. Isolate identifiers beginning with “B”, “R”, and “O” denote isolates derived from backyard flocks, racing pigeons, and ornamental pigeons, respectively. Symbol shapes indicate the host group origin of each isolate: circles represent racing pigeons, squares correspond to ornamental pigeons, and stars denote backyard pigeons. Isolates highlighted in grey correspond to *C. coli*, whereas the remaining isolates are classified as *C. jejuni*. Bootstrap values (based on 1000 replicates) are shown at the nodes and indicate the robustness of the inferred phylogenetic relationships. The outer ring indicates the *flaA* gene allele types associated with each isolate

Diversity analyses corroborated these findings, with Simpson’s Diversity Index (SDI) exceeding 0.90 across all pigeon groups, reflecting extensive allelic variability and a non-clonal population structure. Ornamental pigeon isolates showed the broadest distribution of unique alleles relative to their sample size, whereas racing pigeon isolates tended to cluster around a limited set of dominant alleles, including 93 and 294 (Fig. 1).

### Antimicrobial susceptibility testing

Out of 131 *Campylobacter* isolates tested, AMR was widespread, although resistance patterns varied across antimicrobial classes and pigeon groups. Overall, TE resistance was the most frequently detected phenotype, observed in 84/131 (64.1%) isolates, followed by resistance to CIP in 71/131 (54.2%) isolates and erythromycin (E) in 65/131 (49.6%) isolates. In contrast, CN resistance was markedly lower, detected in 19/131 (14.5%) isolates (Table 4).

**Table 4:** Distribution of *flaA*-SVR alleles among *Campylobacter* isolates from pigeons across different management systems, including previously reported host associations derived from the PubMLST database. The designation “multiple hosts” was applied to alleles reported in more than four distinct host categories in the PubMLST database

Allele	Total (*n* = 131)	Backyard (B)	Racing (*R*)	Ornamental (O)	Previosuly reported host (PubMLST)
93	9	4	5	0	wild bird
199	8	5	2	1	chicken, goose
383	5	1	2	2	wild bird
419	5	1	1	3	multiple hosts
22	5	1	2	2	multiple hosts
67	5	2	1	2	multiple hosts
80	5	2	3	0	wild bird
111	5	3	0	2	cattle
766	5	3	2	0	pig, chicken
262	4	2	1	1	pig, chicken
294	4	1	3	0	environment, meat
58	4	1	1	2	human, chicken
8	4	2	1	1	multiple hosts
10	3	1	0	2	multiple hosts
555	3	3	0	0	duck, human, environment
666	2	2	0	0	multiple hosts
983	3	0	2	1	human, wild bird
1203	3	1	2	0	human, wild bird
14	2	1	1	0	multiple hosts
52	2	1	1	0	multiple hosts
106	2	1	1	0	human
17	2	1	1	0	human, chicken
1376	3	1	1	1	human, chicken
1378	2	1	1	0	human, chicken
156	2	1	0	1	human, pig, cattle
27	2	0	1	1	multiple hosts
964	2	0	2	0	human, pig, chicken
135	2	0	2	0	human, cattle
5	2	0	1	1	multiple hosts
1	2	1	0	1	chicken, human, wild bird
66	2	1	0	1	multiple hosts
11	2	1	0	1	multiple hosts
98	1	1	0	0	chicken
103	1	1	0	0	human
96	1	1	0	0	multiple hosts
100	2	2	0	0	multiple hosts
105	1	1	0	0	multiple hosts
92	1	1	0	0	multiple hosts
82	1	1	0	0	human, chicken
81	1	1	0	0	human, wild bird
442	1	1	0	0	multiple hosts
1609	1	0	1	0	human, chicken, cattle
112	1	0	1	0	human, chicken, turkey
1304	1	0	1	0	chicken
157	1	0	0	1	human, chicken, cattle
444	1	0	0	1	multiple hosts
487	1	0	0	1	human, cattle
303	1	0	0	1	human, chicken
21	1	0	0	1	human, chicken
777	1	0	0	1	human, chicken, cattle

At the group level, backyard pigeons exhibited the highest overall resistance burden, with 42/56 (75.0%) *Campylobacter* isolates resistant to at least one antimicrobial agent. Tetracycline resistance was particularly frequent among strains isolated from backyard pigeons (37/56; 66.1%), alongside high rates of CIP (31/56; 55.4%) and E resistance (29/56; 51.8%). Racing pigeons showed intermediate resistance levels, with 28/43 (65.1%) isolates resistant to one or more antimicrobials; TE (26/43; 60.5%) and CIP (21/43; 48.8%) were the most frequent resistance phenotypes. Ornamental pigeons showed the lowest resistance rates, although resistance to at least one antimicrobial was noted in 20/32 (62.5%) isolates, most frequently to TE (16/32; 50.0%) and CIP (19/32; 59.4%). Significant differences between groups were observed for erythromycin resistance, with backyard pigeons showing lower resistance levels compared to racing pigeons (*p* = 0.005) and ornamental pigeons (*p* = 0.046). Similarly, ciprofloxacin resistance differed significantly between groups, with ornamental pigeons exhibiting lower resistance compared to both racing (*p* = 0.029) and backyard pigeons (*p* = 0.013) (Table 5).

**Table 5:**
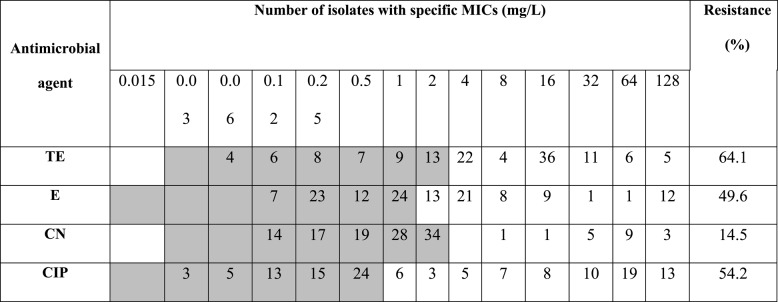
Distribution of Minimum Inhibitory Concentrations (MICs) in *Campylobacter* strains (*N* = 131) isolated from pigeons

Shaded areas indicate the susceptibility range of each antibiotic tested: (*TE*) Tetracycline, (*E*) Erythromycin, (*CN*) Gentamicin, (*CIP*) Ciprofloxacin

Multidrug resistance (MDR) was identified in 29/131 (22.1%) isolates. The majority of MDR isolates originated from backyard pigeons (15/56; 26.8%), followed by racing pigeons (10/43; 23.3%) and ornamental pigeons **(**4/32; 12.5%). Among MDR strains, *C. jejuni* accounted for 21/29 (72.4%) isolates, while *C. coli* represented 8/29 (27.6%) isolates. However, differences in MDR prevalence between groups were not statistically significant (*p* > 0.05).

The most common MDR pattern was TE-CIP-E, detected in 11/29 (37.9%) MDR isolates, followed by TE-CIP-CN (6/29; 20.7%) and TE-E-CN (4/29; 13.8%). A smaller subset of isolates (3/29; 10.3%) exhibited complete resistance to all four tested antimicrobials.

## Discussion

*Campylobacter* spp. are ubiquitous in the environment and have been frequently recovered from a wide range of wild and free-living birds, which are considered important reservoirs and disseminators of the pathogen across ecosystems [10, 11]. The *Campylobacter* prevalence observed in this study (29.1%) aligns with findings from avian reservoirs such as feral pigeons and other synanthropic bird populations. A study of urban pigeon populations in Madrid reported *C. jejuni* carriage in as many as 69.1% of pigeons [74], while pooled estimates across synanthropic pigeon populations are around 24%, with regional variations ranging from 16% to 20% [11, 32, 75]. Meanwhile, a study conducted in urban northern Egypt revealed a *Campylobacter* prevalence of 36.0% via culture-based methods, comprising 20% *C. jejuni* and 16% *C. coli* [76]. These data place the results of the present study within the expected epidemiological framework for pigeons, indicating that semi-open and mobile pigeon cohorts—such as backyard and racing flocks—can mirror or even exceed feral carriage rates in their environments, in contrast to more isolated or captive populations.

Notably, a clear gradient in prevalence emerged across pigeon categories, with backyard flocks showing the highest rates (37.3%), racing pigeons demonstrating moderate levels (28.7%), and ornamental pigeons displaying the lowest carriage (21.3%). In a study conducted in Germany, *Campylobacter* detection rates in racing pigeons ranged from 13.1% to 23.7% per loft, while 40% of lofts tested positive at least once during repeated sampling [77]. By comparison, backyard flocks, which share environmental exposures with domestic livestock, may approach higher carriage frequencies, whereas ornamental pigeons—typically kept indoors with controlled feed and regular cleaning—show patterns that are more consistent with captive or urban populations, where transmission is less pronounced [78].

In terms of species composition, *C. jejuni* was predominant (85.5% of isolates), while *C. coli* accounted for 14.5% of isolates. This mirrors patterns observed in wild bird species, where *C. jejuni* predominance is well established due to its enhanced adaptation to avian gut environments [11, 76]. The higher proportion of *C. coli* in racing pigeon isolates (18.6%) than in isolates from backyard and ornamental pigeons (12.5% each) underscores the potential for mobility-mediated bacterial exchange. Racing pigeons’ exposure to diverse environments, including agricultural areas and natural foraging sites, as well as frequent contact with other flocks may facilitate the acquisition of *C. coli*, which is less common in more contained populations [79, 80].

The virulence gene repertoire of pigeon-derived *Campylobacter* isolates shows broad and heterogeneous distribution, mirroring patterns described in other avian reservoirs while also highlighting host-specific differences. The presence of *flaA* in all isolates reaffirms its role as a highly conserved structural and motility-associated gene, as reported in earlier pigeon and wild bird studies where *flaA* was almost invariably present, reflecting its importance for colonisation and gut persistence [47, 81–83].

Adhesion-related genes were widespread, with *cadF* detected in over 90% of all isolates and particularly prevalent among *C. jejuni*. These findings are consistent with previous studies of avian isolates from Korea and Japan, in which *cadF* and *jlpA* were also identified as dominant adhesion determinants [84, 85]. The fact that 66.4% (87/131) of isolates harboured a complete adhesion gene cluster (*flaA–cadF–jlpA–porA–pebA*) is epidemiologically important, as it indicates that pigeons carry strains equipped with the full genetic repertoire required for efficient adherence to epithelial cells. Similar clustering of adhesion genes has been observed in birds from complex environments, supporting the view that semi-free-ranging pigeon flocks—particularly backyard and racing birds—act as ecological reservoirs of *Campylobacter* with high colonisation potential [10, 85, 86]. Comparable gene distribution patterns have also been described in commercial poultry and waterfowl, where *cadF*, *jlpA*, and *flaA* represent core colonisation determinants across *C. jejuni* isolates [87, 88]. In ducks, high detection frequencies of *cadF* and *porA* have been linked to enhanced mucosal adherence and transmission potential, and similar adhesion profiles have been documented in chickens and turkeys, indicating that these genetic elements are conserved across avian hosts regardless of management system [83, 89].

Invasion-associated genes were also highly prevalent, especially *ciaB* and *pldA*, both of which were significantly enriched in *C. jejuni*. Similar observations were made in domestic geese in Poland, where *pldA* prevalence exceeded 90% of isolates [90]. Interestingly, a substantial proportion of *C. coli* isolates carried invasion determinants (*ciaB* in 57.9%), which contrasts with the generally reduced virulence repertoire reported for *C. coli* in poultry and wild birds [91–93]. This may suggest context-specific horizontal gene exchange, especially in racing pigeons, whose exposure to diverse environments and interspecies microbial communities could promote the acquisition of invasion-related loci [79, 94]. The infrequent detection of *virB11* (8.4%) aligns with previous avian studies, where plasmid-mediated type IV secretion systems are sporadic, often confined to specific ecological niches, and generally considered less important for *Campylobacter* colonisation in avian hosts [48].

The cytolethal distending toxin (CDT) gene cluster (*cdtA–cdtB–cdtC*) was widely distributed, with more than 70% of isolates harbouring the complete operon. This prevalence is strikingly higher than that reported in some wild bird populations, where partial detection of CDT genes is more common [10, 85, 86, 95, 96]. The high prevalence of the complete *cdtABC* cluster among racing pigeon isolates (79.1%) suggests cytotoxic potential in this subgroup. Given the pivotal role of CDT in host DNA damage and cell cycle arrest [97], this finding may reflect both the increased virulence of strains circulating in mobile pigeon populations and their heightened potential as zoonotic reservoirs.

Genes implicated in post-infectious sequelae, notably *wlaN* and *cgtB*, were infrequently detected but occurred at higher frequencies than often reported. In this study, *wlaN* and *cgtB* were detected in 17.6% (23/131) and 15.3% (20/131) of isolates, respectively, and were more prevalent among isolates from racing pigeons. Many avian surveys reported markedly lower detection rates of these loci (often < 5–10%) in non-clinical isolates, which has bolstered the assumption that birds play a minimal role in GBS-associated *Campylobacter* epidemiology [95, 98–100]. The relatively higher prevalence of these loci, noted in the current study, suggests that pigeon-associated strains may occasionally retain or acquire LOS sialylation potential more frequently than expected. Interestingly, *wlaN* was found only in a single *C. coli* isolate, despite the fact that sialylated LOS classes are rare in *C. coli*. This observation raises the possibility of horizontal gene transfer or unique selective pressures within pigeon-associated microbiomes [101]. From a public health perspective, this finding is noteworthy in the context of frequent and close contact between pigeons and humans, particularly hobbyists, breeders, and racing pigeon handlers. Repeated handling, cleaning of lofts, and exposure to pigeon faeces may facilitate the transmission of *Campylobacter* strains harbouring these genetic determinants. Although the overall prevalence of GBS-associated markers remains moderate, their presence in pigeon-derived isolates indicates that even non-clinical avian reservoirs may contribute to the circulation of strains with the potential to induce post-infectious complications in humans, thereby warranting further investigation.

The analysis of the *flaA*-SVR locus revealed considerable genetic heterogeneity among *Campylobacter* isolates from pigeons, consistent with its role as a sensitive marker of microevolutionary dynamics and host–pathogen interactions [102, 103]. In contrast to previous studies of backyard poultry and waterfowl, where novel alleles were frequently reported [47, 82], all alleles identified in the present study were already represented in the PubMLST database, suggesting that pigeon-associated *Campylobacter* populations are not shaped by newly emerging genotypes but by lineages with established ecological distributions.

Nevertheless, the host associations of the identified alleles are noteworthy. Alleles 80 and 93, detected at relatively high frequencies, have previously been reported almost exclusively in wild bird reservoirs, suggesting that racing and backyard pigeons may act as secondary carriers of genotypes originating in migratory avifauna. Similarly, alleles 383 and 766ࣧ restricted to wild birds in earlier reportsࣧwere recovered from multiple pigeon isolates, reinforcing the view that pigeons may act as ecological bridges between feral and synanthropic avian populations. The detection of allele 1203, which is also largely confined to wild birds, further supports this interpretation (PubMLST, accessed in August 2025).

Conversely, several alleles primarily isolated from livestock hosts were also identified among pigeon isolates, particularly those from backyard and ornamental flocks. According to the PubMLST database, alleles 111 and 156, both previously reported from bovine sources in the United States, as well as allele 666, historically linked to cattle, and allele 199, described in chickens and geese, were all detected in pigeon-derived strains. Although the PubMLST dataset does not include detailed publication references for these entries, the available host metadata clearly suggests potential cross-host transmission at the pigeon–livestock interface, where environmental overlap and indirect contact may facilitate genetic exchange. This pattern was most pronounced in backyard pigeons, which showed the broadest range of allele origins in this study, suggesting that their more heterogeneous environmental exposure may facilitate contact with genotypes associated with multiple livestock hosts. These observations indicate that pigeons may act as zoonotic intermediaries, contributing to the maintenance and spread of *Campylobacter* lineages relevant to both animal and human health. Environmental associations were also evident, as allele 294, most frequently reported in surface waters, and allele 555, found in ducks and aquatic environments, were detected in backyard and racing pigeon isolates, pointing to possible waterborne exposure routes in semi-open habitats with access to unprotected water sources. Moreover, allele 262, associated primarily with pigs and only rarely reported in wild birds, as well as the poultry-related allele 1376, previously identified in chickens, were detected in pigeon isolates. Together, these findings highlight the ecological convergence between pigeon populations and domestic livestock reservoirs, as reflected in the PubMLST database.

Beyond the poultry industry, pigeons and other birds have emerged as underestimated factors in the maintenance and dissemination of antimicrobial-resistant *Campylobacter*. The resistance landscape in pigeon-derived isolates aligns with broader patterns reported for avian reservoirs, with TE and fluoroquinolones as the main selective pressures. The proportion of TE-resistant isolates in this study (64.1%) was markedly higher than that reported for wild birds in a large Italian wildlife-rescue cohort, where resistance reached only 17.6% overall [75]. In contrast, CIP (54.2%) and E (49.6%) resistance values were more comparable to those reported in European pigeon and wild-bird populations. For example, CIP resistance was determined at 30–40% in German racing pigeons (Teske, 2013) and at 20–35% in Polish wild ducks and gulls [37]. Similarly, TE resistance rates of only 20–25% were reported in feral pigeons in Spain [74], underscoring the unusually high prevalence observed in the current study. Gentamicin resistance was absent in the Italian wildlife cohort, whereas a prevalence of 14.5% was detected in pigeon isolates in this study, indicating a broader resistance spectrum than is typically reported in non-captive avian surveys.

Pigeon-focused data from Poland similarly indicate substantial resistance burdens. In domestic and free-living pigeons, Dudzic et al. reported approximately 40% tetracycline resistance and widespread resistance to multiple antimicrobial agents, including erythromycin, streptomycin, and ampicillin, with resistance to two or more drugs observed in all tested strains. Although antimicrobial panels differ across studies, tetracycline resistance provides a useful benchmark and highlights that pigeon-associated isolates frequently harbour acquired resistance determinants, which is consistent with the high prevalence of tetracycline resistance and multi-class resistance patterns observed in the present study [104]. In this context, the resistance profiles observed in backyard pigeons are of particular concern, as this group exhibited both the highest frequency and the greatest diversity of resistance determinants. Their close proximity to households, domestic animals, and feed sources creates conditions conducive to the persistence and amplification of antimicrobial-resistant *Campylobacter* strains, as well as their potential spillover to livestock and humans. Importantly, although pigeons are not routinely subjected to antimicrobial treatment, the observed resistance patterns may be driven by indirect selective pressures. These include environmental contamination with antimicrobials originating from agricultural activities, contact with livestock-associated reservoirs, and exposure to manure or contaminated water sources. Furthermore, co-selection mechanisms linked to environmental pollutants, such as heavy metals or disinfectants, may contribute to the maintenance and dissemination of resistance determinants within pigeon-associated bacterial populations [94, 96].

Taken together, these findings indicate that backyard pigeons may represent an important reservoir of antimicrobial-resistant *Campylobacter* in urban and peri-domestic settings. More broadly, the results support the consideration of pigeons as potential sources of antimicrobial-resistant *Campylobacter* for humans, poultry, and livestock. In particular, racing pigeons, due to their long-distance movements and frequent contact with heterogeneous environments, including agricultural areas and natural habitats, may facilitate the acquisition and dissemination of resistant strains across ecological boundaries. Similar interfaces between wild birds, livestock, and the environment have been identified as critical points for pathogen exchange and AMR spread [52, 80, 96]. Moreover, growing evidence indicates that birds can act as vectors of antimicrobial-resistant bacteria across large geographic distances, contributing to the circulation of clinically relevant resistance determinants [10, 30]. In this context, pigeon populations, especially those maintained under semi-open conditions, may function as ecological bridges between environmental, animal, and human-associated reservoirs, thereby reinforcing their relevance within the One Health framework.

At the macro level, the observed phenotype hierarchy — with fluoroquinolone resistance predominating, followed by tetracycline resistance and much lower aminoglycoside resistance — is consistent with global reports on *Campylobacter*, which indicate the highest resistance rates for fluoroquinolones, intermediate levels for tetracycline, and generally low levels for gentamicin [105, 106]. In the present study, the proportion of gentamicin-resistant isolates (14.5%) was higher than that typically reported in wildlife, including wild birds, where resistance is usually below 5% [107]. These findings suggest that pigeon-associated *Campylobacter* populations may reflect resistance patterns commonly described in other reservoirs, while also exhibiting some differences in the distribution of specific resistance traits. However, given the cross-sectional design of the study, no conclusions can be drawn regarding the directionality of transmission or the role of pigeons in the amplification of antimicrobial resistance.

The prevalence of MDR in this study (22.1%) was comparable to wildlife-focused estimates. For example, a review of wild bird studies reported an MDR prevalence of approximately 33% in *Campylobacter*, albeit with substantial heterogeneity across host species, ecologies, and antimicrobial panels [108].

Notwithstanding the comprehensive scope of the present study, several limitations should be considered when interpreting the findings. First, the cross-sectional design precludes assessment of the temporal dynamics and persistence of *Campylobacter* colonisation within pigeon populations. Second, individual-level metadata, including age, clinical history, and prior antimicrobial exposure, were not consistently available, thereby limiting the evaluation of host-associated risk factors. Third, susceptibility to gentamicin was interpreted using Enterobacteriaceae breakpoints in the absence of *Campylobacter*-specific criteria, which may affect comparability with studies applying alternative interpretative frameworks. Another limitation of this study is the targeted focus on *C. jejuni* and *C. coli*, which, although epidemiologically dominant, excludes less frequently reported species such as *C. lari* and *C. upsaliensis*, potentially leading to an underestimation of species diversity within the analyzed population. Finally, although *flaA*-SVR typing constitutes a useful tool for assessing genetic diversity, its discriminatory capacity is lower than that of higher-resolution approaches such as multilocus sequence typing (MLST) or whole-genome sequencing, which could provide more detailed insights into strain-relatedness and transmission dynamics.

Future research should focus on the application of high-resolution genomic approaches, particularly whole-genome sequencing, to further elucidate the phylogenetic relationships, transmission pathways, and genetic determinants of antimicrobial resistance in pigeon-associated *Campylobacter* populations. Such analyses would enable more precise source attribution and improve understanding of strain circulation between wildlife, livestock, and human-associated environments. In addition, longitudinal studies are needed to assess the temporal dynamics of *Campylobacter* carriage in pigeons and to identify key environmental and host-related factors influencing colonisation and persistence. Expanding surveillance to include a broader range of pigeon populations and geographic regions would also contribute to a more comprehensive assessment of their role within the One Health framework.

## Conclusions

The present findings indicate that pigeons represent an important, yet often overlooked, reservoir of *Campylobacter*. The consistently high prevalence of *Campylobacter* spp. in pigeon populations, exceeding that typically reported in wild birds, underscores their role as efficient carriers of zoonotic pathogens [10, 109, 110]. Unlike many wild avian hosts that harbour *Campylobacter* only sporadically, pigeonsࣧparticularly those kept in backyard, ornamental, and racing settingsࣧlive in proximity to humans, thereby amplifying the public health relevance of *Campylobacter* carriage in these populations.

Taken together, the presence of alleles associated with wild birds, livestock, poultry, and environmental sources within pigeon populations highlights their epidemiological relevance and supports their role as reservoirs and potential vectors of *Campylobacter* diversity. By linking allele distribution patterns to historical host associations, these findings suggest that pigeons may not only maintain a distinct *Campylobacter*-specific allele pool but may also indicate potential ecological connectivity between different host-associated reservoirs, although no conclusions regarding direct transmission can be drawn.

The high frequency of virulence determinants in pigeon-derived isolates, combined with a substantial AMR burden, reinforces their potential impact on zoonotic transmission dynamics. Pigeons should therefore be recognised not merely as urban “nuisance birds,” but as relevant actors in the ecology of *Campylobacter*, warranting greater attention in surveillance frameworks. From a public health perspective, these findings encourage greater emphasis on pigeons as a critical and highly interactive host group at the human–animal–environment interface, rather than an exclusive focus on wild birds as presumed primary reservoirs.

## Data Availability

None of the data was deposited in an official repository. All the data obtained in the present research are presented in this manuscript. The data that support the study findings are available from the authors upon request.

## Abbreviations

AMR: Antimicrobial resistance
E: Erythromycin
EUCAST: European Committee on Antimicrobial Susceptibility Testing
Campylobacter spp.: Campylobacter species
C. coli: Campylobacter coli
C. jejuni: Campylobacter jejuni
CIP: Ciprofloxacin
CN: Gentamycin
MDR: Multidrug resistance
MIC: Minimum inhibitory concentration
TE: Tetracycline
